# Expect the Unexpected: Leveraging the Human-Robot Ecosystem to Handle Unexpected Robot Failures

**DOI:** 10.3389/frobt.2021.656385

**Published:** 2021-07-26

**Authors:** Shanee Honig, Tal Oron-Gilad

**Affiliations:** Department of Industrial Engineering and Management, Mobile Robotics Laboratory and HRI Laboratory, Ben-Gurion University of the Negev, Be’er Sheva, Israel

**Keywords:** unexpected failures, human-robot ecosystem, social robots, non-expert user, resilience engineering, resilient robots, user-centered, failure handling

## Abstract

Unexpected robot failures are inevitable. We propose to leverage socio-technical relations within the human-robot ecosystem to support adaptable strategies for handling unexpected failures. The Theory of Graceful Extensibility is used to understand how characteristics of the ecosystem can influence its ability to respond to unexpected events. By expanding our perspective from Human-Robot Interaction to the Human-Robot Ecosystem, adaptable failure-handling strategies are identified, alongside technical, social and organizational arrangements that are needed to support them. We argue that robotics and HRI communities should pursue more holistic approaches to failure-handling, recognizing the need to embrace the unexpected and consider socio-technical relations within the human robot ecosystem when designing failure-handling strategies.

## Introduction

In 2016, a security robot at a shopping mall ran over a child and kept walking (Kircher, 2016). In 2017, a patrol robot rolled itself into a fountain (Swearingen, 2017). In 2020, a delivery robot got stuck on a sidewalk and needed to be rescued (Media, 2020). As robots become more common in public spaces (44% growth in 2019; IFR, 2020), questions of what to do when they fail become increasingly important. In most Sci-Fi movies, when the protagonist’s autonomous tool breaks down, they leave it and move on. In reality, the robot is its owner’s responsibility. Suppose 85-year-old Maggie was at the mall with her robot assistant, when suddenly it ran over a child, fell into a fountain, or got stuck. Maggie cannot ignore the incident, she cannot shut the robot off and leave it at the mall, and she cannot carry it home herself. What should she do?

Despite their grandiose portrayals in the media, robots still struggle to reliably perform tasks in nondeterministic environments. Failures, “degraded states of ability causing the behavior or service being performed by the system to deviate from the ideal, normal, or correct functionality” (Brooks, 2017, p.9), often occur. Countless autonomous fault-diagnosis and failure-handling methods have been developed (Ku et al., 2015; Hanheide et al., 2017; O’Keeffe et al., 2018; Kaushik et al., 2020). Yet, design improvements will never fully eliminate the potential for unexpected failures. Assistive robots will operate in many dynamic unstructured environments, among people with changing goals, abilities, and preferences (Jung and Hinds, 2018), so there will always be unexpected events, challenging the robot (Woods, 2018).

We propose to leverage socio-technical relations within the human-robot ecosystem (HRE) to develop strategies for handling unexpected failures. Social interactions with technical parts of the ecosystem can be the source of unexpected failures (Drury et al., 2003; Carlson and Murphy, 2005; Mutlu and Forlizzi, 2008), help detect unexpected failures (Giuliani et al., 2015; Mirnig et al., 2017) and facilitate resolutions (Steinbauer, 2013; Knepper et al., 2015). For example, bystanders can cause robots to freeze, but can also help robots identify and overcome technical obstacles. Customer service policy can dictate whether unexpected failures are resolved quickly or continue to escalate. Considering unexpected failures within the broader socio-technical ecosystem can predict sources of failure and novel methods of response.

When performance is challenged by extenuating circumstances, organized structures and processes in the HRE will change (Holling, 1996), influencing the robot's ability to respond. For example, if the robot sparked a fire; bystanders, who normally would help the robot, may refuse to assist. Social resources (like emergency services or specialized engineers) may become available to overcome the failure. Therefore, there are bilateral relationships between unexpected robot failures and socio-technical aspects of the HRE; unexpected robot failures can be caused by members of the ecosystem and the robot's response to them can impact the structure of the ecosystem, which, in turn, may influence the robot's ability to further respond. Considering relationships between the robot and its surrounding social context becomes critical for understanding which failure handling strategies are available.

To model socio-technical relations within the HRE, and understand how they impact robots abilities to respond to unexpected failures, we apply the Theory of Graceful Extensibility (TGE; Woods, 2018). TGE explains fundamental principles behind successful cases of sustained adaptability in systems. Sustained adaptability, the “ability to be poised to adapt,” is critical for systems to respond and recover from unexpected disruptions (Woods, 2019). Most theoretical frameworks assume that certain components in the ecosystem remain fixed, focus on specific types of socio-technical relations or do not consider systemic changes that occur when the ecosystem is significantly challenged (e.g. Zieba et al., 2011; Ouedraogo et al., 2013; Ruault et al., 2013). TGE is flexible enough to account for different socio-technical relations, recognizing that entities within the ecosystem and their relations are constantly changing. It provides insight regarding how the ecosystem’s structure influences its ability to adapt to surprise events, like unexpected failures.

Prior studies viewed robots as part of larger technological ecosystems (Quan and Sanderson, 2018; Kim, 2019) or performed socio-technical analyses (Fiore et al., 2011; Lima et al., 2016; Shin, 2019; Morgan-Thomas et al., 2020). However, studies on the impact of social infrastructure on robots’ ability to handle unexpected failures are scarce (Honig and Oron-Gilad, 2018). Viewing human-robot relations within a socio-technical ecosystem using TGE offers new directions for research. In advancing this perspective, we respond to calls to identify new strategies for predicting, preventing and handling unexpected robot failures (Zhang et al., 2017). We highlight and demonstrate first and second order relations between the robot’s response to unexpected failures and the socio-technical ecosystem it resides in, frequently ignored in robotics literature. Our goal is to trigger the robotics and HRI communities to adopt holistic approaches to failure-handling, recognizing the need to embrace the unexpected and consider socio-technical relations within the HRE.

## The Theory of Graceful Extensibility

TGE provides a formal base for characterizing how complex systems maintain or fail-to-maintain adaptability to changing environments, contexts, stakeholders, demands, and constraints. Complex systems are modeled by “Tangled Layered Networks” of adaptive units (UABs; Units of Adaptive Behavior). Each UAB has adaptive capacity - the potential for adjusting activities to handle future changes. This generates a range of adaptive behaviors allowing the UAB to respond to changing demands (termed Capacity for Maneuver; CfM). Since this range is finite, all UABs risk saturation (running out of CfM) when presented with surprises (events that fall near or outside boundaries). Consequently, units require ways to extend their adaptive capacity when they risk saturation.

Performance of any UAB as it approaches saturation differs from when it operates far from saturation, resulting in two forms of adaptive capacity: base and extended. Base adaptive capacity refers to the potential to adapt to well-modeled changes (far from saturation) and is more ubiquitous in contemporary robotic design. In this mode, the goal is efficiency (“faster/better/cheaper”). Extended adaptive capacity, or Graceful Extensibility, represents the ability to expand CfM when surprise events occur (when risk of saturation is increasing or high). Near saturation, UABs aim to maintain performance.

Layers in the network represent hierarchical functional relations between UABs - lower layers provide services to upper layers (Doyle and Csete, 2011). In networks with high graceful extensibility, UABs in upper layers continuously assess the risk of saturation of themselves, their neighbors, and UABs in lower layers, by monitoring the relationship between upcoming demands and response capacity. When risk is high, upper UABs act to increase CfM of lower UABs by changing priorities, invoking new processes, extending resources, removing potential restrictions, empowering decentralized initiatives, and rewarding reciprocity. UABs increase the CfM of their neighbors by providing assistance (e.g., sharing resources). A UAB’s ability to model and track CfM is limited, and their localized perspective in the network obscures their perceptions of the environment, so ongoing efforts and shifts in perspective are required to improve a unit’s estimation of its own and others’ capabilities and performance.

Woods (2018) identified three common patterns leading adaptive systems to break down. Decompensation, exhausting the capacity to adapt as challenges grow faster than solutions can be implemented, occurs when CfMs of UABs are mismanaged, UABs are not synchronized, or lower level UABs are unable to take actions to limit event escalation. Working at cross purposes occurs when one UAB increases its CfM while reducing CfM of others, i.e., locally adaptive but globally maladaptive, and is often caused by mis-coordination across UABs. The third pattern involves failing to ensure current strategies are still effective as system states change. To sustain adaptability, one should empower decentralized initiative at lower layers, reward reciprocity and coordinate activities between UABs to meet changing priorities.

## Applying the Theory of Graceful Extensibility to Failure Handling of Assistive Robots

The HRE’s ability to adapt and respond to unexpected failures can be modeled via TGE. Since we aim to improve service and understand socio-technical relations, we take a systems approach. We view the robot as one adaptive unit within a broader system, rather than delving into architectural components, consistent with other socio-technical networks (Mens, 2016). For clarity of presentation, we layered UABs by their ability to extend the CfM of lower UABs, rather than by their hierarchical functional relations. Although the models are abstracted representations of HREs, they are sufficient to showcase the importance of socio-technical relations to the design of failure-handling strategies for unexpected robot failures.

### Base Adaptive Capacity

Imagine that a sensor on Maggie’s robot malfunctioned while at the mall, and the robot no longer recognizes obstacles or people in its environment. The robot runs its automatic diagnostic program, failing to find anomalies. Neither the robot nor Maggie know what caused the issue or how to resolve it. Maggie wants to complete her shopping, return home with her robot and belongings, and fix the malfunction as quickly as possible. The robot is carrying Maggie’s belongings, some of which she cannot carry herself.

**FIGURE 1:**
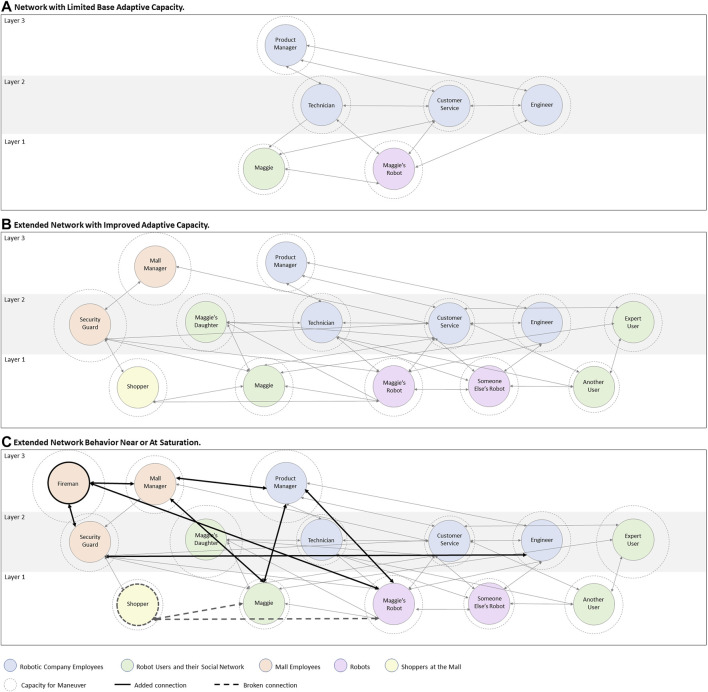
Tangled Layered Networks of socio-technical relations in the Human-Robot Ecosystem (HRE), **(A)** with limited base Adaptive Capacity, **(B)** with improved base Adaptive Capacity, **(C)** adapting in response to an unexpected event that challenged the network.

One possible network is modeled in Figure 1A. This network is representative of many robotic services today, which rely primarily on communications between the robot, the user and the service provider (through the robot, customer service, technicians or online) to resolve failures. Each UAB has its own CfM, indicated by the size of the dotted circle around it. Links between UABs are depicted by arrows. An engineer can send a push update to Maggie’s robot (extending its CfM), so it is in a higher layer than the robot.

Various social and socio-technical strategies for resolving the unexpected issue are supported by this network. For example, the robot could ask Maggie to guide it through a safe path with no obstacles (e.g., by holding its hand) and display a warning signal to alert people in the environment to stay clear. The robot could automatically flag the problem to an engineer to remotely run debugging tests; or to customer service, who could initiate a service call to help Maggie resolve the issue. The robot could guide Maggie through questions to help isolate the problem source.

Unexpected failures fall within base adaptive capacity when the existing HRE infrastructure is able to successfully help UABs adapt to achieve their goals efficiently, while managing their risk of saturation. Here, the sensor failure would fall within base adaptive capacity if the type of coordination needed to enable Maggie to complete her shopping and return home in a timely manner is well-supported within the ecosystem (e.g., if customer service managed to remotely fix the sensor failure, or if Maggie was able to guide the robot back home safely, despite the sensor failure).

The base adaptive capacity of this network is quite limited, as communications are distributed across few UABs. If the connections within the HRE were extended (e.g., in Figure 1B), various additional social and socio-technical strategies could be leveraged to resolve a wider variety of unexpected issues, increasing the base adaptive capacity of the network. For example, expert users could be rewarded for providing troubleshooting support to Maggie. The robot could call a security guard to move Maggie’s belongings to a shopping cart and store the robot until a technician arrives. Robots of the same model could help it identify the issue; sharing the outcomes of their learning algorithms or prior experiences (Arrichiello et al., 2017). The robot could call Maggie’s daughter to pick them up and wait for a technician elsewhere. If the socio-technical infrastructures that are needed to support these interactions have been put into place in advance, then the risk of saturation for Maggie, the robot, customer service agents, and other UABs in the network would remain low despite the unexpected issue.

The robot’s technical design will influence the ability of solutions to take place. For the security guard to take the robot to storage, it must be easily moveable. For Maggie’s daughter to pick up her mother and the robot, it must be light enough to be lifted into a car. For inexperienced users to help the robot identify and/or resolve unknown issues, there must be a user-friendly interface that guides them. Social and socio-technical failure-handling solutions require social and technical infrastructures to support them.

### Behavior Near or At Saturation

Imagine that Maggie's robot accidentally sparked fire at the mall. How will the network in Figure 1B respond? If the robot alerts people who are already aware of the fire or are not receptive to listening, or starts its usual diagnostics while ignoring the fire altogether, it is failing to ensure its current strategies are still effective. The robot can alert an engineer, who can then alert the product manager, initiating crisis management processes, but by the time this feedback loop closes, the situation in the mall may escalate. This would be an example of decompensation. The robot can attempt to direct people toward an emergency exit, however, if it is programmed to walk at slow speed, it may block other escapees. This would be an example of the robot working at cross purposes.

Maggie’s goal would no longer be to return home quickly with her belongings and the robot, but to return home safely. To provide good service in such situations, the entire ecosystem will adapt in unexpected ways to meet this new goal. For example, the robot may need to obtain permission to walk faster than its normal speed range. An engineer previously working on developing new features, may need to send the robot’s last location to emergency services. Customer service agents may need to contact owners of similar robots to prevent additional occurrences.

Examples of possible ad-hoc network changes due to the fire are modeled in Figure 1C in bold. These changes can impact the ability of UABs to further respond to this event. For example, if firefighters are using the robot to locate the fire, it may not be able to lead Maggie to an emergency exit. If the call center is overwhelmed by concerned customers, Maggie may not be able to reach them to explain how the fire began. New resources will become available, e.g., firefighters can locate and/or extinguish the fire. Contrarily, some resources in the ecosystem that under normal circumstances are leveraged, may no longer be available, e.g., people at the mall may become reluctant or unavailable to help. The CfM of UABs in the network could change based on changes to the network that occurred as a result of the failure–for example, the product manager’s CfM could increase as a result the fire, because they would be justified in setting aside all non-urgent responsibilities and using/recruiting additional resources to respond to the extenuating circumstances. It is therefore important for the robot and service providers to consider the immediate consequences of attempted failure-handling measures and the unintentional outcomes that occur due to socio-technical changes within the ecosystem.

### Graceful Extensibility

#### Preparing for Unexpected Robot Failures That Challenge the Ecosystem

While it is impossible to eliminate the risk of robotic failures or to predict how the HRE will change during surprise events, it is possible to design robots that promote graceful extensibility; allowing the HRE to make required adjustments to accommodate new contingencies. Various strategies can be developed, in advance, to manage risk of saturation and increase the range of adaptive behaviors for better preparedness to unexpected failures. In Table 1, we describe principles based on TGE that support better sustained adaptability in the HRE, and specify recommendations for how robot developers, designers and robot companies can act on them. The result is a set of guidelines for what could be done, in advance, to provide better handling of unexpected robot failures.

**TABLE 1:** Socio-technical considerations for handling unexpected failures that challenge the human-robot ecosystem.

Principle	Research Directions and Recommendations	Related Literature
Monitor and enhance the range of adaptive behaviors (CfM) that units in the HRE can perform.	Incorporate an understanding of the roles and capabilities of people within the HRE into the robot's cognitive architecture to inform decision making and task sharing. Model how human-related, task-related, robot-related, and environment-related factors can impact roles and capabilities.	Scerri et al. (2003), Howard and Cruz (2006), Alami et al. (2011), Fiore et al. (2011), Akkaladevi et al. (2016), Joshi and Sabanovic (2017), Musić and Hirche (2017), Ranz et al. (2017), Welfare et al. (2019), Strohkorb Sebo et al. (2020)
*Description:* Model and estimate CfM of the relevant units in the network (robots, support teams, local support, etc.).	Estimate or obtain information about individuals’ capabilities (e.g., physical limitations, expertise, and perceived mental model) to inform the robot's decision-making.	Arboleda et al. (2020), Milliez et al. (2014), Rossi et al. (2017), Martins et al. (2019), Martín et al. (2020), Umbrico et al. (2020)
Continuously look for ways to enhance capacity (via design or organizational changes). Anticipate bottlenecks and create processes in advance to accommodate them. Share CfM-related information between units in the network.	Incorporate recognition of changes in the social environment (e.g., social activity) into the robot's cognitive architecture. For example, note how the social environment may impact the robot and how subsequent robot behaviors may further impact the social environment (e.g., relations between pedestrians’ flow and robot navigation, robot use and work practices, etc.)	Eldridge and Maciejewski (2005), Lee et al. (2012), Giuliani and Knoll (2013), Jiang et al. (2016), Jiang et al. (2020), Nikolaidis et al. (2017), Chen et al. (2018), Jung and Hinds (2018), Pelikan et al. (2018), Sebo et al. (2020)
*Purpose:* Understand when units may require additional resources, from whom they can ask for help, when they should offer support to others, and when to reprioritize goals. Early detection and prevention of decompensation within the network.	Promote the ability to form accurate mental models of robot behavior and capabilities for people in the HRE (e.g., using shared cognition models).	Marble et al. (2004), Murphy and Burke (2005), Bradshaw et al. (2009), Scheutz et al. (2017), Hellström and Bensch (2018), Kwon et al. (2018), Thomas (2019), Ehsan et al. (2019), Demir et al. (2020), Tabrez et al. (2020), Han et al. (2021), Ramaraj (2021)
	Develop mechanisms for robots to understand and react to emotions, like pain, fear, panic, confusion, and distress, to correctly identify warning conditions requiring a reaction.	Kulic and Croft (2007), Leite et al. (2013), Le and Lee (2014), Jung (2017), Bagheri et al. (2020), de Kervenoael et al. (2020), Spezialetti et al. (2020), Weidemann and Rußwinkel (2021)
	Perform task analysis, communication analysis, workflow analysis, and usability studies of robot interactions with people in the HRE (users, bystanders, customer service agents, etc.). Test in real-world environments, under normal and degraded conditions, to identify challenges, potential degraded states, and conflicts originating from interactions within the HRE. Identify metrics for acceptable levels of service for different levels of degradation.	Casper and Murphy (2002), Casper and Murphy (2003), Burke et al. (2004), Adams (2005), Mutlu and Forlizzi (2008), Cunningham et al. (2013), Sabanovic et al. (2014), Frennert et al. (2017), Welfare et al. (2019), Niemelä et al. (2019), Cameron et al. (2021)
	Improve anomaly recognition of human and robot activities.	Panangadan et al. (2004), Zweigle et al. (2013), Hornung et al. (2014), Häussermann et al. (2015), Ordóñez et al. (2015), Bezemskij et al. (2016), Rossi et al. (2018), Guo et al. (2018), Park et al. (2019), Azzalini et al. (2020), Basurto et al. (2020), Zhou et al. (2020), Castellano-Quero et al. (2021)
	Monitor resources available to robots and other units in the HRE. Identify in advance resource reserves that can be called upon during surprise events (e.g., non-critical robot processes that can be postponed, personnel that can be pulled from their normal assignments to respond to robotic failures, etc.)	Dressler and Fuchs (2005), Sadrpour et al. (2013), De Carolis et al. (2014), Lee (2018), Baums (2019)
	Monitor the time it takes for problems to be solved, the time between surprise events, and the performance level that can be restored following different types of failures to detect decompensation.	Itti and Baldi (2005), Schreckenghost et al. (2009b), Schreckenghost et al. (2009a), Schreckenghost et al. (2010), Cabal-Yepez et al. (2012), Duff et al. (2014), Jiang et al. (2017), Khaldi et al. (2017), Damacharla et al. (2018), Qiao and Weiss (2018)
Reevaluate and reprioritize goals and resources in response to surprise events.	Enable robots to estimate or inquire about people’s changing goals, expectations, mental state, and priorities. Then, adapt robot goals and behaviors accordingly.	Demiris (2007), Breazeal et al. (2009), Gillain et al. (2013), Devin and Alami (2016), Shevtsov and Weyns (2016), Leite et al. (2018)
*Description:* Reevaluate goals and resources when unexpected failures occur. Detect events that fall outside of *base adaptive capacity*.	Develop adaptable emergency response capabilities for robots.	Ostergaard et al. (2001), Kumar et al. (2004), Liu et al. (2007), Ferranti and Trigoni (2008), Witkowski et al. (2008), Tang et al. (2016), Nadi and Edrisi (2017), Sakour and Hu (2017), Hashemipour et al. (2018), Willms et al. (2019)
*Purpose:* Ensure the planned or re-planned course of action is still appropriate.	Develop algorithms optimized to promote operation and adaptation during degraded conditions and surprise events. Prioritize failure-handling solutions that can adapt to facilitate a broad range of failure situations over failure-specific solutions.	Zhang et al. (2017), Chattunyakit et al. (2019), Tan et al. (2020), Wilson et al. (2020)
	Develop adaptive resource allocation and management strategies.	Mainland et al. (2005), Airy et al. (2009), Berenz and Suzuki (2011), Eibel et al. (2015), Zhang et al. (2016), Afrin et al. (2019), Afrin et al. (2021), Trucco et al. (2021)
Facilitate coordination and synchronization between units in the HRE.	Design robots to facilitate and encourage bi-directional communication between people in the HRE (e.g., the robot can provide users and bystanders with relevant contacts for troubleshooting and emergencies, give recommendations on when and where to find help, facilitate direct calls to the support team, etc.)	Yamamoto et al. (1992), Park et al. (2009), Kristoffersson et al. (2013), Kramer and Demaerschalk (2014), Nyssen and Blavier (2017), Hock et al. (2018)
*Description:* A broad range of communication and coordination types should be supported, and redundancy is encouraged. When possible, units in the HRE should be encouraged and empowered to consider a situation from another unit's perspective. Communication channels should support bi-directional messaging between units in the ecosystem.	Share failures and efforts to relieve them between stakeholders in the HRE in a way that optimizes their ability to understand the situation and contribute to solutions (e.g., by including all relevant information, by preventing negative emotional responses like panic or stress, etc.)	Engelhardt et al. (2017), Honig and Oron-Gilad (2018), Nayyar and Wagner (2018), Sebo et al. (2019), Banerjee et al. (2020), Cameron et al. (2020), Choi et al. (2020), Kontogiorgos et al. (2020), Tolmeijer et al. (2020), Washburn et al. (2020)
*Purpose:* to prevent decompensation, to prevent working at cross purposes, and to empower decentralized initiative.	Encourage shifts in perspective. Develop robots that can imagine the world from another viewpoint, leveraging the perspectives views of users, bystanders, and other agents to clarify potential ambiguities, inform decision-making and resolve unexpected issues.	Trafton et al. (2005), Breazeal et al. (2006), Warnier et al. (2012), Pandey and Alami (2013), Pandey and Alami (2014), Milliez et al. (2014), Milliez et al. (2016), Fischer and Demiris (2016), Zhao et al. (2016)
	Develop inter-firm networks and standardized protocols to ensure different robotic frameworks can communicate, cooperate and learn from one another.	Mizukawa et al. (2000), Bruyninckx (2001), Das et al. (2005), Ferketic et al. (2006), Schilling and Phelps (2007), Lechevalier et al. (2011), Spulber (2013), Foucart and Li (2021)
Enable and encourage units in the HRE to help one another.	Design approachable robots that provide people with advice, feedback and instructions in a manner that encourages cooperation and compliance, particularly during first encounters (e.g., by adhering to social and cultural norms).	Yamamoto et al. (1992), Torrey (2009), Wang et al. (2010), Torrey et al. (2013), Trovato et al. (2013), Salem et al. (2014), Geiskkovitch et al. (2016), Gehle et al. (2017), Choi et al. (2019), Xu (2019), Hashemian et al. (2019), Sanoubari et al. (2019), Lim et al. (2020), Avelino et al. (2021), Ullrich et al. (2021), Fischer et al. (2021), Tian and Oviatt (2021)
*Description:* Units should be actively encouraged to take initiative in helping other units resolve problems. Motivating mechanisms and incentives must be formed.	Develop mechanisms to help robots identify when people may require additional support and when the robot should offer help or take initiative.	Torrey et al. (2013), Ramachandran et al. (2016), Baraglia et al. (2017), Brscic et al. (2017), Ito et al. (2020)
*Purpose:* Reward reciprocity, prevent decompensation and empower decentralized initiative.	Design mechanisms and interaction technologies that enable robots to ask for and receive human assistance effectively from bystanders and inexperienced users in degraded conditions. Identify when it is appropriate to ask for help, whom the robot may ask, and what type of assistance can be expected. Test robot designs under degraded conditions.	Huttenrauch and Eklundh (2003), Ross et al. (2004), Hüttenrauch and Severinson-Eklundh (2006), Thomaz and Breazeal (2007), Rosenthal et al. (2011), Rosenthal et al. (2012), Wongphati et al. (2012), Foster et al. (2013), Fischer et al. (2014), Glas et al. (2015), Bajones et al. (2016), Cha and Mataric (2016), Srinivasan and Takayama (2016), White et al. (2020), Ito et al. (2020)
	Develop mechanisms that enable robots to help repair one another and/or facilitate mutual learning and problem solving (viewing individual robots as part of a distributed team).	Bererton and Khosla (2001), Bererton and Khosla (2002), Burke and Murphy (2007), Kutzer et al. (2008), Davis et al. (2016)

## Discussion

Existing robotic failure-handling techniques struggle to create appropriate responses to predictable failures, let alone unexpected ones that challenge the Human-Robot Ecosystem (HRE). The Theory of Graceful Extensibility provides a framework within which the HRE and its ability to adapt to unexpected failures can be modeled, evaluated, and improved. By expanding from Human-Robot Interaction (HRI) to HRE, adaptable failure-handling strategies can be identified, alongside social and technical infrastructure requirements needed to support them.

Investing in responses to unexpected failures is a fine balancing act (Woods, 2018). Resources that improve performance near saturation may undermine performance far from saturation. Encouraging people to contact customer service will inevitably lead to increased demand for customer service, costing money that could have gone toward improving the robot’s failure-prevention systems. Similarly, resources that support graceful extensibility challenge the ecosystem's desire for efficiency during normal operations. Sustained adaptability requires the ecosystem to continuously search for the balance between improving base adaptive capacity and supporting graceful extensibility.

The ability to handle unexpected failures requires shared acknowledgement that unexpected failures are natural and unavoidable. Robotic companies today seem to prioritize perception of perfection over facilitating open communication and collaboration within their ecosystems. It is currently difficult to obtain information regarding the types of failures robots experience and possible resolutions. This is problematic for all failure types, but particularly for unexpected ones, as it often takes additional effort to differentiate between known and unknown problems. Grassroots efforts to overcome poor socio-technical relations such as YouTube instructional videos are less effective as complexity increases. Forewarning people of robot imperfections can improve evaluations of the robot and quality of service following failures (Lee et al., 2010). Taking steps to improve communication, cooperation, and collaboration between people in the ecosystem is likely to improve customer acceptance of robots.

Issues of information overload, control management and privacy arise from many of the strategies suggested to support adaptive capacity. How do we facilitate communication and collaboration between people in the ecosystem without annoying or overwhelming them? Who decides with whom the robot can share information and what it can share? How do we protect Maggie’s privacy? Many social, legal and ethical questions are raised from this approach and remain unresolved (Scheutz and Arnold, 2016; Santoni de Sio and van den Hoven, 2018). Close collaborations with government agencies, regulators, and related service corporations and organizations (e.g., malls, hospitals, etc.) will be needed to answer these questions. However, we strongly believe in the importance of extending adaptive capacity through socio-technical means in order to handle unexpected failures in robots.

## Data Availability

The original contributions presented in the study are included in the article/supplementary material, further inquiries can be directed to the corresponding author.
